# Large‐Aperture Polarization‐Independent Broadband Achromatic All‐Dielectric Metalens for Terahertz Focusing

**DOI:** 10.1002/advs.74515

**Published:** 2026-02-17

**Authors:** Xiaoqiang Jiang, Xu Chen, Zechuan Bin, Fu Tang, Min Hu, Manman Li, Wenhui Fan, Baoli Yao

**Affiliations:** ^1^ State Key Laboratory of Ultrafast Optical Science and Technology Xi'an Institute of Optics and Precision Mechanics Chinese Academy of Sciences Xi'an China; ^2^ Aviation Key Laboratory of Science and Technology on Advanced Surface Engineering AVIC Manufacturing Technology Institute Beijing China; ^3^ Terahertz Research Center School of Electronic Science and Engineering University of Electronic Science and Technology of China Chengdu China; ^4^ University of Chinese Academy of Sciences Beijing China

**Keywords:** achromatic, imaging, large‐aperture, metalenses, terahertz

## Abstract

As a promising alternative to conventional lenses, terahertz (THz) achromatic metalenses with planar configurations are of great significance in broadband optical applications. However, as the application scenarios extending to macro‐scale far‐field optical imaging, an inherent conflict arises between the increasing demand for large‐aperture, high focusing efficiency, broadband achromatic metalenses and the steep rise in design complexity and fabrication costs. Herein, a multifunctional achromatic metalenses design scheme is proposed to realize large‐aperture, high focusing efficiency, and polarization‐insensitive THz all‐dielectric single‐layer metalenses, by integrating the discrete multi‐wavelength achromatic design method and the global optimization algorithm. The designed metalens is successfully fabricated with a diameter up to 2.21 cm, which is the largest reported THz achromatic metalens to our knowledge. Experimental measurement results demonstrate that it has polarization‐insensitive characteristics and excellent achromatic focusing performance from 0.80 to 1.20 THz, with achromatic coefficient values below 2.9% and average focusing efficiencies greater than 46% under both *x*‐ and *y*‐polarized incident waves. Furthermore, the broadband achromatic imaging performance is characterized and validated, confirming its remarkable achromatic imaging capability. This study not only showcases an outstanding THz achromatic metalens but also presents a generalizable design strategy applicable across the entire electromagnetic spectrum.

## Graphical abstract

A large‐aperture, high focusing efficiency and polarization‐independent THz single‐layer achromatic metalens is reported via discrete multi‐wavelength achromatic design and global optimization algorithm. The fabricated metalens exhibits a largest diameter of 2.21 cm, excellent achromatic performance, extremely high average focusing efficiency (> 46% under *x*‐ and *y*‐polarization incidence), and remarkable broadband achromatic imaging capability.

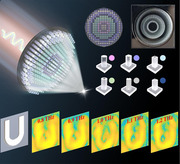

## 1 Introduction

Terahertz (THz) waves, occupying the unique spectral range from 0.1 to 10 THz, are extensively utilized in the fields including spectroscopy, imaging, communication, and biomedicine [1, 2, 3, 4]. Over the past decades, the pursuit of advancing THz technology has motivated considerable efforts on high‐power THz sources and high‐sensitivity detectors, generating numerous significant achievements [5, 6]. However, owing to the weak electromagnetic (EM) response, strong dispersion, and absorption loss of natural materials at THz frequencies, the shortage of components is becoming a critical barrier to the development of THz technology [7]. Metasurfaces, composed of subwavelength meta‐atoms, have emerged as a novel class of optical materials to precisely manipulate the amplitude, phase, and polarization of EM waves [8, 9, 10, 11, 12]. The development of metasurfaces offers a promising pathway to establish a transformative platform for designing compact and multifunctional THz devices with customized spectral responses [13, 14, 15, 16, 17, 18, 19, 20, 21, 22].

Among the various applications of THz technology, imaging has emerged as a particularly active research area, as evidenced by numerous fundamental studies and industrial applications [23, 24, 25, 26]. As a critical component of a THz imaging system, the performance of lenses is a primary determinant for the overall image quality. Motivated by the requirements of high‐resolution imaging and non‐destructive inspection, the compact, high‐frequency and high focusing efficiency THz lenses become a critical priority for far‐field imaging applications. However, traditional THz focusing components, such as spherical convex and TPX lenses, are typically bulky and suffer from inherent issues, including significant aberrations, low efficiency, and insufficient resolution, which fundamentally limit their employment in THz imaging systems. Unlike traditional bulky lenses, metalenses can arbitrarily engineer their phase profiles and modify the properties of EM waves, providing an effective solution for the development of compact and multifunctional THz lenses. From visible light to microwaves, various metalenses with diverse functionalities have been proposed and attracted significant research interests [27, 28, 29, 30, 31]. Despite the unparalleled advantages and flourishing prospects, the practical application of metalenses still faces challenges such as limited aperture size and inferior performances in focusing efficiency, achromatic bandwidth, and numerical aperture (NA). It should be noticed that the aperture size of traditional lenses in current optoelectronic systems is at least centimeter‐scale, which is much larger than most reported metalenses. The aperture disparities inevitably impede modern optical systems from fully exploiting the planarity and lightweight characteristics of metalenses. As a long‐term pursuit goal in meta‐devices, the large‐aperture metalenses can overcome issues of insufficient imaging area and effectively match with far‐field imaging systems [32]. However, the development of large‐aperture metalenses is confronted with substantial challenges, such as excessively large layout data, high fabrication costs, and prohibitive computational requirements.

Numerous investigations have been dedicated to addressing the aperture limitation of metalenses. In 2022, Zhang et al. fabricated a near‐infrared metalens for space telescope with diameter of 80 mm by using pattern splicing and wafer rotation method [33]. In 2024, Hou et al. developed a joint stepper lithography method to realize a 5 cm diameter metalens at 10.6 µm for a thermographic camera [34]. By implementing a deep‐ultraviolet lithography process that stitched multiple exposure fields with different reticles, Park et al. demonstrated a 100 mm diameter metalens at 632.8 nm for cosmos imaging [35]. Despite significant progress in large‐aperture metalenses, chromatic aberration—causing wavelength‐dependent focal shifts and image quality degradation—remains a major obstacle for broadband applications. Several pioneering works, primarily in the visible and infrared regimes, have been proposed to eliminate chromatic aberrations by spatial multiplexing, dispersion engineering, and optimization algorithms [36, 37, 38, 39, 40, 41]. Nevertheless, nearly all reported achromatic metalenses are limited to millimeter‐scale and struggle to simultaneously achieve high focusing efficiency and large NA due to the inherent trade‐offs among aperture size, efficiency, working bandwidth, and NA [38, 42]. Therefore, simultaneously achieving a large‐aperture, high efficiency, and achromatic functionality in a single‐layer metalens remains a critical challenge. This is particularly pronounced in the THz band, where such devices are seldom reported [43, 44, 45, 46], especially for achromatic metalenses possessing large‐aperture, high focusing efficiency, and polarization‐insensitive characteristics.

**FIGURE 1:**
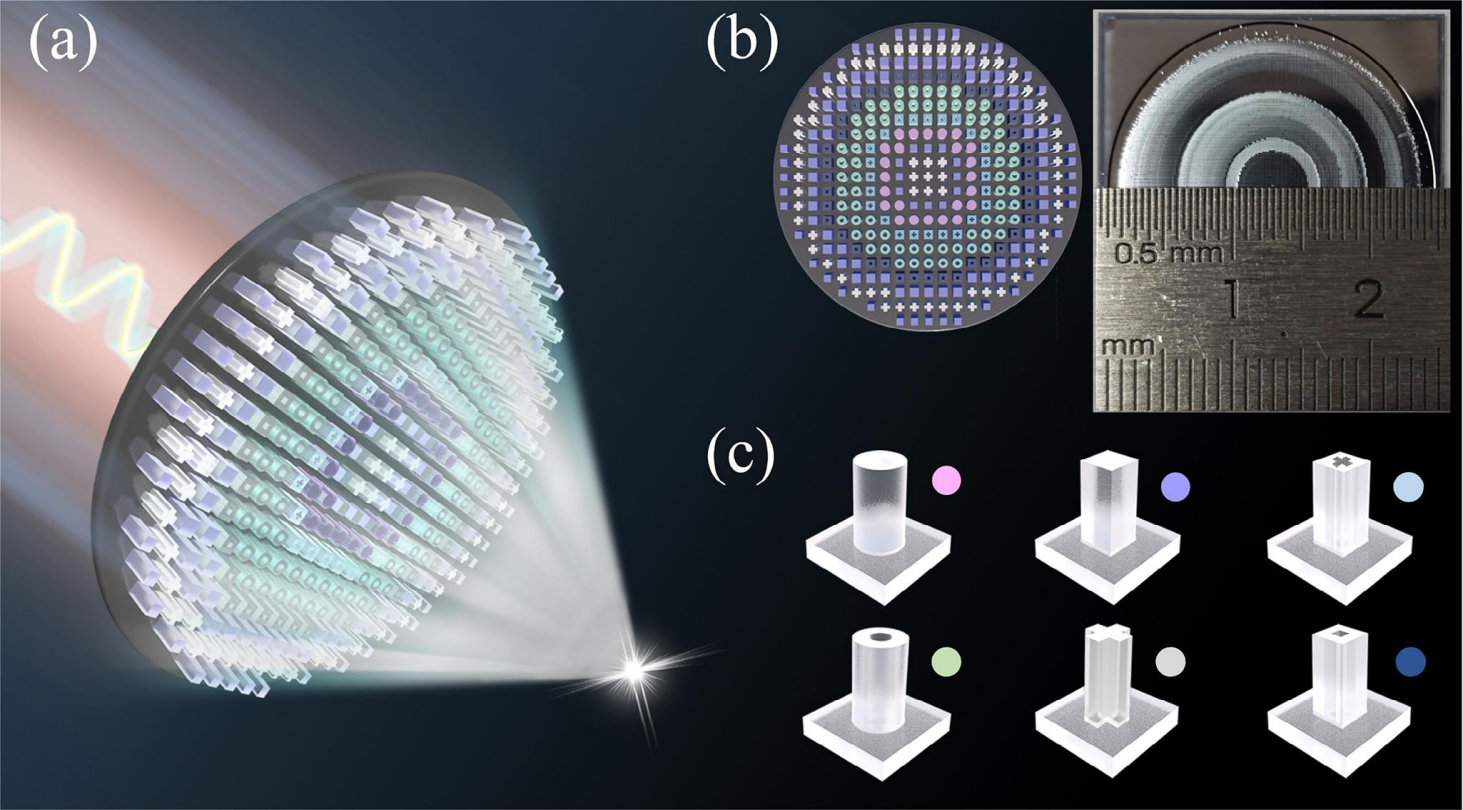
(a) The schematic diagram of the designed LAPIAM. (b) The aperture size of LAPIAM. (c) Six different shapes of rotationally symmetric meta‐atoms.

In this work, a novel approach is proposed and experimentally demonstrated for single‐layer large‐aperture and polarization‐insensitive achromatic metalens (LAPIAM) in the THz domain, as illustrated in Figure 1a. Six rotationally symmetric meta‐atoms with distinct geometries are designed and simulated to modulate the amplitude and phase of THz waves, thereby constructing a meta‐atom library with full 2π phase coverage. A combined strategy utilizing a discrete multi‐wavelength achromatic design and particle swarm optimization (PSO) algorithm is employed to globally optimize the hyperbolic focusing phase of achromatic metalens at nine sampling frequency points. The designed all‐dielectric metalens is fabricated and experimentally characterized, with a diameter up to 2.21 cm, representing the largest reported THz achromatic metalens to date. Experimental measurement results demonstrate that the proposed LAPIAM achieves excellent broadband achromatic focusing from 0.80 to 1.20 THz, with achromatic coefficient values below 2.9% and average focusing efficiencies greater than 46% under both *x*‐ and *y*‐polarized incident waves. Furthermore, the broadband achromatic imaging performance of LAPIAM is also investigated, demonstrating its remarkable capability for THz achromatic imaging. The proposed LAPIAM not only paves the way for replacing conventional THz lenses and advancing integrated imaging systems but also establishes a versatile design paradigm applicable across the entire EM spectrum.

## 2 Results and Discussion

### 2.1 Design Principle and Method

For broadband achromatic metalenses, the aperture size is primarily constrained by phase dispersion compensation range of the meta‐atoms, which introduces the inherent trade‐offs among NA, bandwidth, and compensation phase imposes a theoretical maximum on the aperture size, as discussed in Section S1. Additionally, conventional single meta‐atom size optimization inherently restricts design freedom, making it difficult to simultaneously satisfy phase distribution and polarization conversion efficiency and ultimately forcing a trade‐off between focusing efficiency and achromatic performance. To overcome these inherent issues and realize a large‐aperture, high focusing efficiency broadband achromatic metalens, the discrete multi‐wavelength design strategy combined with particle swarm optimization (PSO) algorithm is proposed, where the designed metalens achieves broadband focusing, and its aperture size reaches 2.21 cm, as shown in Figure 1b. The meta‐atoms are displayed in Figure 1c, which consists of six high aspect ratio and rotational symmetry structures with the cross‐sectional shape being circle, ring‐shaped, cross‐shaped, cross hole‐shaped, square, and square hole‐shaped, respectively. Due to their four‐fold rotational symmetry in the *x*‐*y* plane, the meta‐atoms exhibit polarization‐insensitive EM responses. Moreover, the meta‐atom library composed of six distinct meta‐atoms provides a significantly expanded select space, which enables the PSO algorithm to select a meta‐atom at each position optimally matching the ideal multi‐frequency phase profile, thereby minimizing the overall wavefront error and maximizing the overall lens efficiency. By fully utilizing their structural degrees of freedom, the designed meta‐atoms can overcome the trade‐off between the polarization conversion efficiency and the dispersion group delay with the jointly modulating geometric phase and transmission phase, thus realizing the target focusing phase of the achromatic metalens [47, 48]. In the simulation and experiment, high‐resistance silicon is exploited as the building blocks for meta‐atoms due to its non‐dispersive refractive index (∼3.45) and low absorption loss in the THz band. Besides, an all‐silicon structure is compatible with existing semiconductor manufacturing techniques and does not require additional film deposition processes, effectively reducing fabrication costs.

**FIGURE 2:**
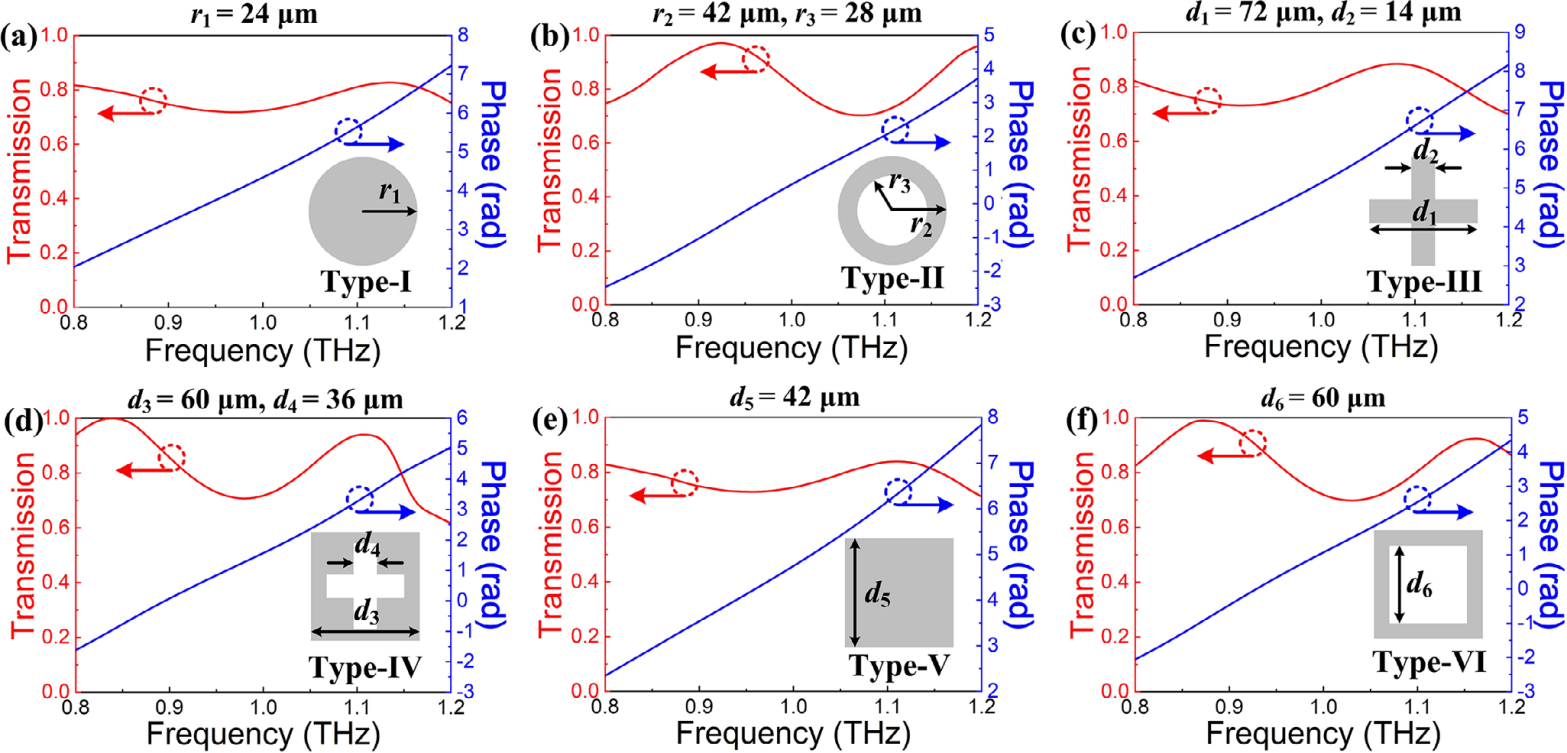
The transmission and phase responses of six meta‐atoms. (a) Circle, (b) Ring‐shaped, (c) Cross‐shaped, (d) Cross hole‐shaped, (e) Square, and (f) Square hole‐shaped.

To illustrate the control capability of the meta‐atoms on amplitude and phase, the Finite‐Difference Time‐Domain (FDTD) method is performed to simulate the EM response of the meta‐atoms, where *x* and *y* directions are set as periodic boundary conditions and *z* direction is set as a perfect matched layer. The optimized lattice constant and height of the meta‐atoms are set as *p* = 100 µm and *H* = 240 µm, respectively. The EM responses of six different meta‐atoms under *x‐*polarization incident waves are depicted in Figure 2, all of which have transmission higher than 70% and also exhibit a large range of phase coverage over 0.8 to 1.2 THz. To further reveal the confinement ability of the meta‐atoms to the incident wave, the magnetic field distribution is simulated and shown in Figure S1. It is observed that the incident wave can be entirely confined inside the meta‐atoms, implying that crosstalk and coupling effects between adjacent structures can be ignored and the phase modulation for each meta‐atom will not be affected in further arrangements.

Due to the constraints in processing capability and manufacturing accuracy, the maximum achievable aspect ratio of the designed silicon meta‐atoms is about 20:1. Consequently, the minimum line width of 12 µm and the maximum size of 84 µm are set to avoid coupling effects between adjacent structures. By changing the radii *r* or side lengths *d* of the six meta‐atoms, the meta‐atoms library of the designed metalens can be constructed, which includes transmission and phase responses simulated under *x*‐polarization incident waves at nine sampling points (frequency interval of 0.05 THz) among 0.80 to 1.20 THz. The sweeping ranges of structural parameters for six types of meta‐atoms are discussed in Section S3.

**FIGURE 3:**
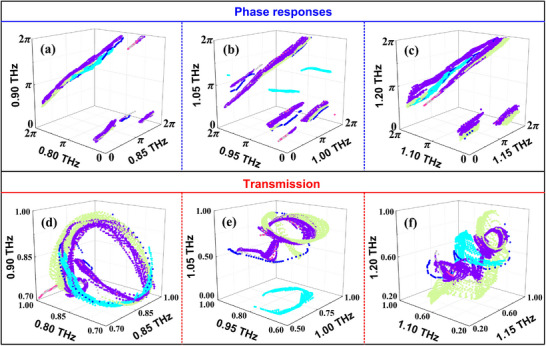
The phase (a–c) and transmission (d–f) response values of the six types of meta‐atoms at nine sampling frequencies from 0.80 to 1.20 THz, with the data for each type displayed in different colors.

According to the scanning range and step size of the meta‐atoms, there are 12286 data of transmission and phase response values for each sampling frequency point. The EM responses of different meta‐atoms at nine discrete points are plotted and divided into three groups for clear presentation. Figure 3a shows the phase responses at frequencies of 0.80, 0.85, and 0.90 THz, where the six different colored dots represent the phase values of the corresponding meta‐atoms. Figure 3b,c also show the phase values at 0.95 1.00, 1.05, 1.10, 1.15, and 1.20 THz, respectively. It can be evidently observed that the meta‐atoms can achieve complete 0 to 2π phase coverage at all sampling frequency points, providing a solid foundation to satisfy the complicated phase requirements. Similarly, the transmission values of six types of meta‐atoms at nine sampling frequency points are simulated and shown in Figure 3d–f, demonstrating the overall high transmission across the entire operating frequency band.

To converge broadband incident THz waves at target position, the metalens should simultaneously satisfy the hyperbolic focusing phase at nine sampling points according to the discrete multi‐wavelength achromatic design method. Therefore, the target phase of each sampling frequency point needs to be corrected by adding an additional phase term and expressed as [49]:

(1) $$\varphi_{ideal}\left(x,y,f\right)=-\frac{2\pi f}{c}\left(\sqrt{x^{2}+y^{2}+{F_{l}}^{2}}-F_{l}\right)+C\left(f\right)$$

where *c* is the speed of light in the vacuum, *f* represents the working frequency, *F_l_* is the designed focal length, and *C*(*f*) is the additional phase term only related to the working frequency.

To show the effect of *C*(*f*) on focusing phase shift, the distribution of focusing phase at 0.80, 1.00, and 1.20 THz is plotted, as shown in Figure S2. It can be observed that the *C*(*f*) can introduce an additional degree of freedom by permitting longitudinal shifts of the spectral phase curves, which improves phase matching with the meta‐atom library and removes the constraint of global linear phase‐frequency dependence. In contrast to the continuous wavelength achromatic design method, this approach simplifies the design complexity by discretizing the 0.80 to 1.20 THz range into nine frequency points, thereby avoiding the necessity for a linear phase‐frequency relationship. And the bandwidth and achromatic performance can be greatly enhanced by increasing the number of discrete sampling points. However, it should be noticed that the discrete design inevitably introduces a discrepancy Δ*φ*(*x*, *y*, *f*) between the actual phase *φ_real_* (*x*, *y*, *f*) and the target phase *φ_ideal_* (*x*, *y*, *f*) profiles. According to the Rayleigh criterion, if the maximum difference between the actual wavefront and the ideal wavefront is less than a quarter of a wavelength, the actual wavefront can be considered nearly perfect. Therefore, the physical challenge of phase optimization can be transformed into a mathematical optimization problem of minimizing the wavefront phase difference function Δ*Φ_d_*, which can be expressed as:

(2) $$\Delta \Phi_{d}=\left[\sum_{i}\sum_{k}{\left|\varphi_{real}\left(x_{i},y_{i},f_{n}\right)-\varphi_{ideal}\left(x_{i},y_{i},f_{n}\right)\right|}^{2}\right]\;/\left(n_{i}\times n_{k}\right)$$

where *f_n_* is the focal length at optimization points, *n_i_* represents the number of meta‐atoms within the diameter of the metalens, and *n_k_* is the number of sampling frequency points.

Subsequently, the PSO algorithm is employed to solve the minimum value of Δ*Φ_d_*, and its detailed workflow is discussed in Section S5. By combining the discrete multi‐wavelength achromatic design method with the PSO algorithm, the additional phase profile *C*(*f*) and the optimal focusing phase at each sampling frequency point can be obtained. The phase difference between the actual and target profiles continuously decreases with increasing iterations of the PSO algorithm, and it is converged after 600 iterations (Figure S3b). As shown in Figure S4, the actual phase profile at each frequency closely matches the target phase across all nine sampling points. The corresponding meta‐atom geometry and dimensions at each spatial position are determined based on the optimized phase mapping, as arranged in Figure S5. In the calculation, the PSO optimization is configured with a search dimension of 9 and a parameter space of 12286 per frequency, and it is performed on an i9‐13900K CPU with approximately 60 h. Eventually, the LAPIAM composed of 221 × 221 meta‐atoms with the diameter *D* = 2.21 cm, focal length *F_l_* = 8 cm, and operating frequency range of 0.80 to 1.20 THz is accomplished.

### 2.2 Performance Characterization of the Metalens

**FIGURE 4:**
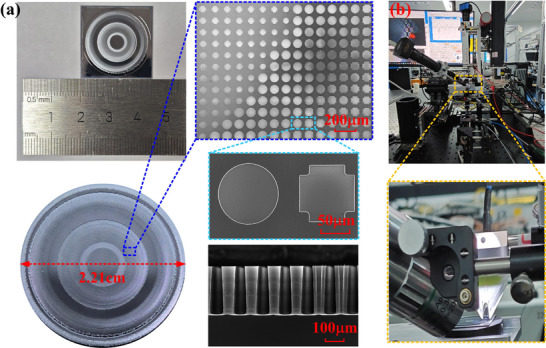
(a) The photograph and the SEM graph of the fabricated metalens. (b) THz near‐field scanning microscopy system.

To demonstrate the performance of the designed metalens, the ultraviolet (UV) lithography and deep silicon etching process is used to fabricate it, with the flowchart shown in Figure S6. Figure 4a depicts the fabricated metalens, which has a diameter of 2.21 cm. The structural morphology of the fabricated metalens is almost identical to the design one, and there is no interconnection between the meta‐atoms. The height of the fabricated meta‐atoms micropillar is approximately 230 µm, exhibiting 4.17% relative error compared with the designed value of 240 µm. Due to the high aspect ratio, the sidewall of the micropillar become inclined during the etching process. These geometric imperfections in the fabricated sample will lead to a discrepancy between the actual and designed focal lengths, reduce the focusing efficiency, and degrade the quality of the focal spot. By combining lithographic overlay technology with deep silicon etching Bosch process and balancing the etch phase and passivation phase, it is possible to fabricate the desired metalens consisting of high aspect ratios micropillar with smooth surfaces, straight sidewalls, and nice alignment [50, 51]. A THz near‐field scanning microscopy (TNSM) system is employed to experimentally characterize the fabricated metalens, as shown in Figure 4b, and the working principle of the experimental system is described in Section S9.

Due to its large‐aperture size and intricate meta‐atom geometry, the direct simulation and calculation of the EM response of LAPIAM requires substantial computational resources and times. To efficiently handle this problem, the FDTD method is first used to calculate the near‐field distribution of the metalens, and subsequently the corresponding far‐field distribution at any position (*z* > *λ*) away from the metalens can be obtained via the Fresnel–Kirchhoff diffraction integral and Fourier transform [52]:

(3) $$u\left(x,y,z;\lambda \right)=\frac{e^{ikd}}{i\lambda d}\;\int \int \;u\left(x_{0},y_{0},z_{0};\lambda \right)e^{\frac{ik}{2d}\left[{\left(x-x_{0}\right)}^{2}+{\left(y-y_{0}\right)}^{2}\right]}dxdy$$

where *d* = *z* – *z*_0_ is the spatial position of the observation surface, *λ* is the working wavelength, and *k* = 2*π*/*λ* is the wavenumber.

**FIGURE 5:**
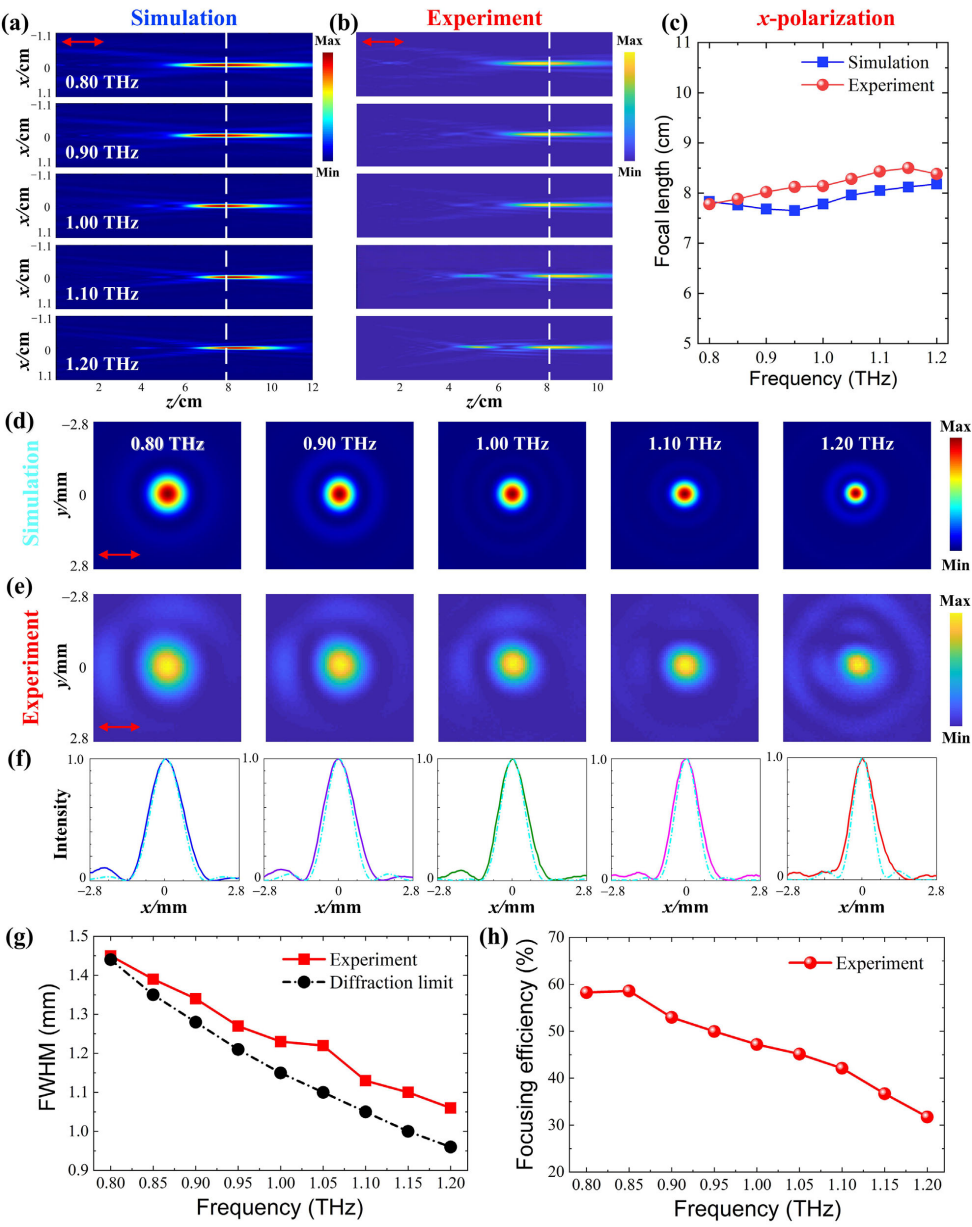
The electric field intensity distribution at *x‐z* plane under *x*‐polarized incident THz waves: (a) Simulation results. (b) Experiment results. (c) Focal length. The electric field intensity distribution at *x‐y* plane: (d) Simulation results. (e) Experiment results. (f) The intensity distribution along *x*‐direction. (g) The measured FWHM. (h) The measured focusing efficiency.

In the experiment, the achromatic focusing performance of LAPIAM is measured under both *x*‐ and *y*‐polarization THz waves by rotating the metalens by 90° between the measurements, where the spatial resolution of the probe during testing is 0.1 mm. Figure 5a,b shows the simulated and experimental intensity profiles along the transmission direction after passing through the LAPIAM. It can be observed that the focal lengths of LAPIAM, identified by the point of maximum intensity in the *x‐z* plane, are barely deviated in the concerned frequency range under *x*‐polarization incidence, confirming its effectiveness in eliminating chromatic aberration over 0.80 to 1.20 THz. Besides, at frequencies of 1.10 and 1.20 THz in Figure 5b, one can see another weak focal point before the focal spot, which arises from a more severe mismatch between the designed and actual phase profiles and the fabrication imperfections. Figure 5c shows the focal length at each sampling frequency, and the average focal length for the experiment and simulation is 8.17 and 7.89 cm, respectively, corresponding to the measured NA of 0.13. It is worth mentioning that the measured NA is relatively small, which is due to the limitation of the phase dispersion control capability in this single‐layer metalens, and makes it unable to achieve high NA while simultaneously achieving large‐aperture, broadband achromatic and high focusing efficiency. The focal length discrepancy between simulation and experiment can be attributed to meta‐atoms parameter deviations, fabrication tolerances, and experimental errors. Moreover, the measured average focal length exhibits a close match to the design focal length (*F_l_* = 8.00 cm), with a relative error of only 2.13%. This result demonstrates that the LAPIAM can effectively converge broadband THz waves into the target position and maintain its achromatic functionality.

To quantitatively evaluate focal length deviation and overall performance of the metalens, the achromatic coefficient value (ACV) is employed as a key metric, and defined as [53]:

(4) $$ACV=\frac{SD}{MV}\times 100\%=\left[\sqrt{\frac{1}{n}\sum_{i=1}^{n}{\left(f_{i}-MV\right)}^{2}}/\frac{1}{n}\sum_{i=1}^{n}f_{i}\right]\times 100\%$$

where SD (standard deviation) and MV (mean value) respectively represent the overall standard deviation and average value of the focal length at nine sampling frequencies.

According to Equation 4, the measured and simulated ACVs of 2.86% and 2.32%, respectively, are both below the 5% threshold for achromatic devices [54]. This result validates the effective chromatic aberration correction of LAPIAM across the 0.80 to 1.20 THz band. From Figure 5d,e, the symmetric focal spots can be observed in both simulation and experiment, accompanied by concentric diffraction fringes that arise from the periodicity of the meta‐atom lattice, which is one‐third of the operating wavelength. Figure 5f shows the electric field intensity at the focal points along *x*‐axis, with a typical Gaussian distribution. Besides, the full width at half maximum (FWHM) of the focal points at sampling frequencies is calculated in Figure 5g. It can be observed that the FWHMs at these sampling frequencies are all close to the Abbe diffraction limit (0.5*λ*/NA), indicating that the LAPIAM has great potential for high‐resolution THz imaging. As a critical performance parameter of the metalens, the focusing efficiency is also investigated and defined as the ratio of the power at the focal spot within the circle of a diameter of 3 × FWHM to the total incident power on the metalens [55]. Figure 5h presents the experimentally measured focusing efficiency at each sampling frequency, with an average value of 46.97% calculated within the working band. It should be noticed that this value is higher than those reported in prior works [43, 45, 49, 56, 57, 58, 59] and is the highest value among polarization‐independent THz achromatic single‐layer metalens to our knowledge. In addition, the simulated FWHM and the focusing efficiency are also shown in Figure S8. One can see that the measured average efficiency is lower than that in simulation, which can be attributed to the fabrication imperfections (deviation of micropillar height, sidewall inclination and collapse of partial meta‐atoms) and measurement errors (inhomogeneity and non‐perfect signal‐to‐noise ratio of the THz source).

**FIGURE 6:**
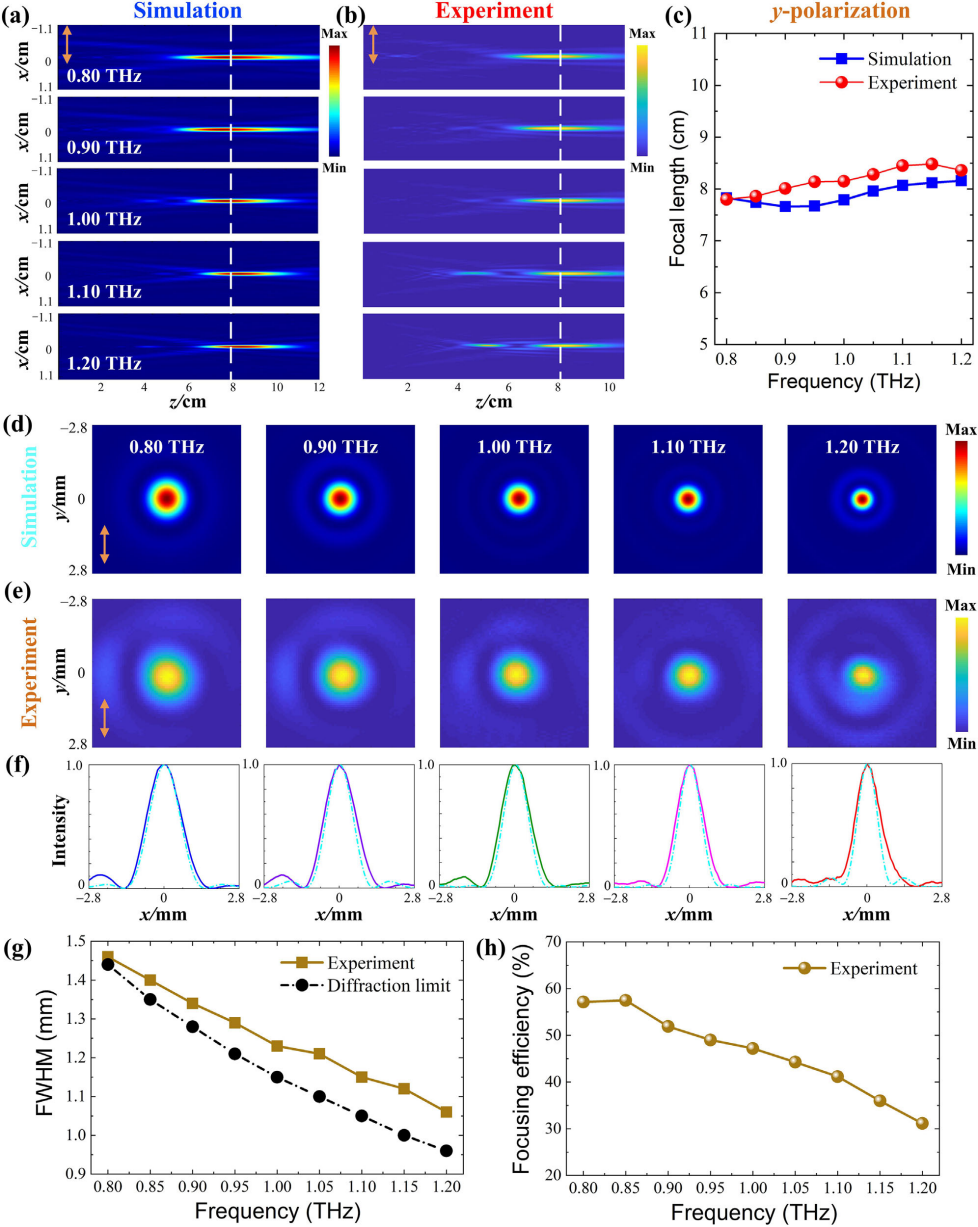
The electric field intensity distribution at the *x‐z* plane under *y*‐polarization incident waves: (a) Simulation results. (b) Experiment results. (c) Focal length. The electric field intensity distribution at the *x‐y* plane: (d) Simulation results. (e) Experiment results. (f) The intensity distribution along the *y*‐direction. (g) The measured FWHM. (h) The measured focusing efficiency.

To demonstrate the polarization‐independent characteristic of LAPIAM, the focusing performance under *y*‐polarization incident waves is also investigated and shown in Figure 6a,b. The focal length of all the sampled frequency points ranging from 0.80 to 1.20 THz is nearly constant, indicating that LAPIAM can also accomplish achromatic functionalities under *y*‐polarized incident waves. To quantitatively elucidate the achromatic performance, both experiment and simulation results of focal lengths at each sampling frequency point are plotted in Figure 6c. The corresponding average focal lengths of experiment and simulation are calculated to be 8.17 and 7.89 cm, respectively, with the measured NA of 0.13. The relative error between the experimental focal length and the designed focal length is only 2.13%, indicating that the metalens can also converge *y*‐polarization THz waves into the target position. Furthermore, the measured and simulated ACVs are 2.84% and 2.24%, respectively, both substantially below the standard threshold and confirming the excellent broadband achromatic performance. Similarly, both simulation and experiment results of the transmitted intensity at the focal plane under *y*‐polarization waves exhibit symmetrical circle spots, as shown in Figure 6d,e. The field intensities of the simulation and experiment at the focal plane along the *x*‐axis also exhibit a typical Gaussian distribution in Figure 6f. In addition, the measured FWHM at each sampling frequency is calculated in Figure 6g, which is also close to the Abbe diffraction limit. Figure 6h shows the experimentally measured focusing efficiency at each sampling frequency under *y*‐polarized incident waves, and its average focusing efficiency is calculated to be 46.14%. The simulated results of the FWHM and the focusing efficiency are also shown in Figure S9. Accordingly, the almost identical focusing performance (e.g., focal spot profiles, focal lengths, FWHMs, and focusing efficiencies) under *x*‐ and *y*‐polarizations observed throughout the operating band confirms the polarization‐independent characteristic of the LAPIAM.

To highlight the advantages of our proposed design method and meta‐devices, a performance comparison with other representative broadband achromatic metalenses in the THz band is listed in Table 1. Previous THz achromatic metalenses designed by the continuous‐wavelength method are confronted with severe aperture constraints (typically millimeter‐scale) due to the competing demands of a linear phase‐frequency response, NA, bandwidth, and phase compensation. In contrast, by integrating a discrete multi‐wavelength achromatic design with a global optimization algorithm, our work overcomes the mutual restrictive relationship among large‐aperture, high focusing efficiency, and broad bandwidth, enabling the realization of a THz achromatic metalens with previously unachievable performances in aperture size, focusing efficiency, and polarization‐independent characteristic simultaneously. Since the complex phase requirements and the limited phase dispersion capabilities of the meta‐atoms, the achromatic bandwidth of our metalens is not particularly wide, which can be further increased by utilizing advanced design methods such as cascaded structures and advanced etching process to enhance the aspect ratio and reduce the inclination of the sidewall of meta‐atoms.

**TABLE 1:** Comparison between our work and previous achromatic metalenses in the THz band.

Refs.	Working range [THz]	Design method	Aperture size [mm]	Focusing efficiency [%]	Polarization characteristics	Numerical aperture
[44]	0.3–0.8	Dispersion phase compensation	10	30–68	Polarization‐ dependent	0.385
[49]	0.9–1.4	Dispersion phase compensation	6.48	26.1 (average value)	Polarization‐ dependent	0.187
[56]	0.75–1.0	Nonlinear phase profile	4	45(maximum value)	Polarization‐ independent	0.384
[57]	0.4–0.8	Dispersion phase compensation	7.2	30 (average value)	Polarization‐ dependent	0.338
[58]	1.0–1.6	Dispersion phase compensation	5.91	26.1 (average value)	Polarization‐ dependent	0.29/0.23
[43]	2.29–2.70	Dispersion and amplitude modulation	10.22	13.01–32.60	Polarization‐ independent	0.125
[59]	2.22–2.86	Group delay dispersion control	6.36	26.87–49.84	Polarization‐ dependent	0.4
Our work	0.8–1.2	Discrete multi‐wavelength and PSO algorithm	22.1	46 (average value)	Polarization‐ independent	0.13

### 2.3 The Achromatic Imaging Performance of LAPIAM

**FIGURE 7:**
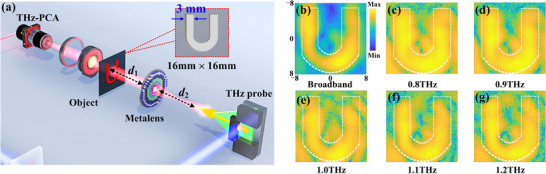
Broadband achromatic imaging experiment. (a) The imaging system. (b) The measured intensity image under broadband THz wave incidence. (c–g) The measured intensity images at 0.8, 0.9, 1.0, 1.1, and 1.2 THz, respectively, the white dashed line representing the outline of the sample.

To further characterize the broadband achromatic imaging performance of LAPIAM, an imaging system based on TNSM is established, as shown in Figure 7a. The imaging objects are 3 mm‐thick U‐shaped metal sheets with a gap of 1.8 and 3 mm, respectively. To obtain an image with equal size of the U‐shaped pattern, the object‐image relationship requires satisfying the standard lens equation of 1/*f* = 1/*d*_1_ + 1/*d*_2_, where *f* = 8.17 cm is the measured focal length and *d*_1_ = *d*_2_ = 16.34 cm are object distance and image distance, respectively. The intensity distribution of the sample with a 3 mm gap is measured under broadband THz wave incidence, and the U shape can be distinctly observed in Figure 7b. Moreover, the intensity distribution from 0.80 to 1.20 THz with 0.10 THz increment is measured and a multispectral imaging is realized. Figure 7c–g shows the transverse intensity distribution across the gap at each frequency, and the U shape can be distinctly identified, and there is no significant discrepancy at each frequency point. While the resolution limit is dependent on the wavelength, the image of the sample can be clearly recorded at all frequencies without tuning the metalens and the target, confirming the broadband achromatic imaging capability of our metalens. For the sample with 1.8 mm gap, it can be identified at broadband and low frequencies while cannot be identified at high frequencies, as shown in Figure S10. It is important to note that there is a significant discrepancy between the actual imaging resolution obtained by experimental system and the resolution calculated based on the measured NA. This discrepancy primarily stems from the severely decreased focusing efficiency and THz power at high frequencies, as well as the experimental error in the imaging system with the separation between the THz probe and the metalens being four times the focal length, thus inevitably introducing the wavefront diffraction.

## 3 Conclusion

In summary, a large‐aperture polarization‐independent achromatic metalens (LAPIAM) was achieved through a synergistic combination of discrete multi‐wavelength achromatic design and particle swarm optimization algorithm. A library of six high aspect ratio and rotationally symmetric meta‐atoms was engineered to achieve complete 0 to 2π phase coverage while preserving polarization‐insensitive operation, substantially expanding the available design freedom. Through discrete sampling at nine frequencies across 0.80 to 1.20 THz and global optimization of phase profiles, a LAPIAM with the largest aperture of 2.21 cm was successfully fabricated. The experimental and simulation results both confirmed that the proposed LAPIAM has excellent achromatic performance with achromatic coefficient values less than 2.9% and average focusing efficiencies greater than 46% under both *x*‐ and *y*‐polarization illumination. Furthermore, the broadband achromatic imaging performance of LAPIAM was also investigated, demonstrating a remarkable THz achromatic imaging capability. This work presents a viable strategy to replace traditional THz refractive lenses and advance the integration of THz systems, showing considerable promise for applications in non‐destructive testing and biomedical imaging.

## 4 Experimental Section

### 4.1 Sample Fabrication

The detailed fabrication process is schematically illustrated in Figure S6, and mainly divided into three parts: (1) Wafer cleaning: In this part, the high‐resistance silicon wafer was performed to double‐sided polishing and cleaning, and then thermally baked at 200°C in Nitrogen gas to remove surface moisture and contamination. (2) Chromium deposition and Photoresist spin coating: A chromium film was deposited on the surface of the cleaned silicon wafer using an electron beam evaporation instrument. Then, the photoresist layer was spin‐coated on the chromium film surface, and subsequently the sample was placed on a heating plate to allow the solvent in the photoresist to evaporate. (3) UV lithography and etching: A metal chromium mask plate was fabricated based on the layout file of the metalens. The sample was then exposed through UV lithography to define the features of each structure. Then, the exposed sample was immersed in the developing solution to reveal the patterns. After that, the patterns were transferred to the hard mask layer by deep reactive ion etching, and the sample with the patterned hard mask was etched. To fabricate the high aspect ratio meta‐atoms, multiple rounds of etching steps were required. The final sample was obtained by removing the patterned hard mask and the small amount of residual chromium, followed by cleaning with the mixed solution of concentrated sulfuric acid and hydrogen peroxide.

### 4.2 Numerical Simulation

The designed metalens is composed of the optimized meta‐atoms automatically determined by a global optimization algorithm and numerically simulated by the FDTD method, where the metalens is placed on *z* = 0 with the *z*‐axis as the optical axis. The Fresnel–Kirchhoff diffraction integral is also employed to characterize its performance. With the open boundary condition being applied, broadband plane waves of *x*‐ and *y*‐polarization respectively illuminated the metalens and then was simulated to characterize its near‐field EM distribution and polarization‐independent characteristic. Subsequently, the Fresnel–Kirchhoff diffraction integral was employed to further evaluate the far‐field performance.

### 4.3 Experimental Measurements

The THz near‐field scanning microscopy (TNSM) system was employed to experimentally characterize the achromatic focusing performance of the metalens, in which the experimental system is shown in Figure S7. In the setup, the THz wave is emitted from a photoconductive antenna and impinges vertically on the sample with a linear polarization. A fiber‐coupled THz near‐field probe was used as the detector, which was mounted on a translation stage to allow 3D scanning of the electric field distribution. The system offers an effective spectrum range from 0.1 to 2.5 THz and a minimum spatial resolution of 20 µm. In addition, the imaging system based on the TNSM system was employed to characterize achromatic imaging performance of the metalens, where the imaging object is placed behind the metalens, and the object distance and image distance were set as equal values to obtain an image with an equal size of the object.

## Conflicts of Interest

The authors declare no conflict of interest.

## Supporting information




**Supporting File**: advs74515‐Sup‐0001‐SuppMat.docx.

## Data Availability

The data that support the findings of this study are available from the corresponding author upon reasonable request.
