# Supplementary material for: Large‐Aperture Polarization‐Independent Broadband Achromatic All‐Dielectric Metalens for Terahertz Focusing
Source: Adv Sci (Weinh). 2026 Feb 17;13(25):e74515. doi: 10.1002/advs.74515 (PMC13137821; doi:10.1002/advs.74515)
Supplement: Supplementary file 1 — Supporting File: advs74515‐Sup‐0001‐SuppMat.docx. [file ADVS-13-e74515-s001.docx]

Supporting Information

Large-Aperture Polarization-Independent Broadband Achromatic All-Dielectric Metalens for Terahertz Focusing

Xiaoqiang Jiang^†^, Xu Chen^†^*, Zechuan Bin, Fu Tang, Min Hu, Manman Li, and Baoli Yao*

X. Jiang and X. Chen contributed equally to this work.

*Corresponding author. Email: chenxu@opt.ac.cn (X. Chen), yaobl@opt.ac.cn (B. Yao)

This Supporting Information file includes:

Supplementary Section 1: The theoretical limit of the radius for continuous wavelength achromatic metalenses

Supplementary Section 2: The magnetic field distribution of the meta-atoms

Supplementary Section 3: The structural parameter variation range of the meta-atoms

Supplementary Section 4: The optimal design method of the achromatic metalens

Supplementary Section 5: The workflow diagram of PSO algorithm and the convergence curve

Supplementary Section 6: Target phase optimization at the sampling frequencies of the metalens

Supplementary Section 7: The meta-atoms arrangement diagram of the metalens

Supplementary Section 8: The fabrication process of the metalens

Supplementary Section 9: The working principle of THz near-field scanning microscopy system

Supplementary Section 10: The simulated performance of the metalens under *x*-polarized incident THz waves

Supplementary Section 11: The simulated performance of the metalens under *y*-polarized incident THz waves

Supplementary Section 12: The achromatic imaging for sample with 1.8 mm gap

**Supplementary Section 1:** **The theoretical limit of the** **radius for continuous wavelength achromatic metalenses**

Conventional single lenses based on refractive and diffractive optics intrinsically suffer from chromatic aberrations, thus limiting their performance in broadband imaging applications. As governed by the generalized Snell’s law [1], the metalens can control the deflection direction of electromagnetic wave thus achieving achromatic focusing. However, the aperture size of the continuous wavelength achromatic metalenses is limited by compensation phase range of the meta-atoms and expressed as [2]:

$R_{\max}\leq\frac{c\cdot\Delta\Phi}{\Delta\omega\left[\frac{1}{NA}-\sqrt{\left(\frac{1}{NA}\right)^{2}-1}\right]}$ (S1)

where *R*_max_ is the maximum achievable radius of the metalens, *c* represents the speed of light in the vacuum, Δ*ω* is the working bandwidth, and ΔΦ is the product of the dispersion range and the working bandwidth. The premise of **equation S1** is that the phase distribution of the generated wavefront meets the requirement of aberration free metalens. It can be seen that the aperture size of the continuous wavelength achromatic metalens is limited by its NA, working bandwidth, and the compensation phase range provided by the meta-atoms.

**Supplementary Section 2: The magnetic field distribution of the meta-atoms**


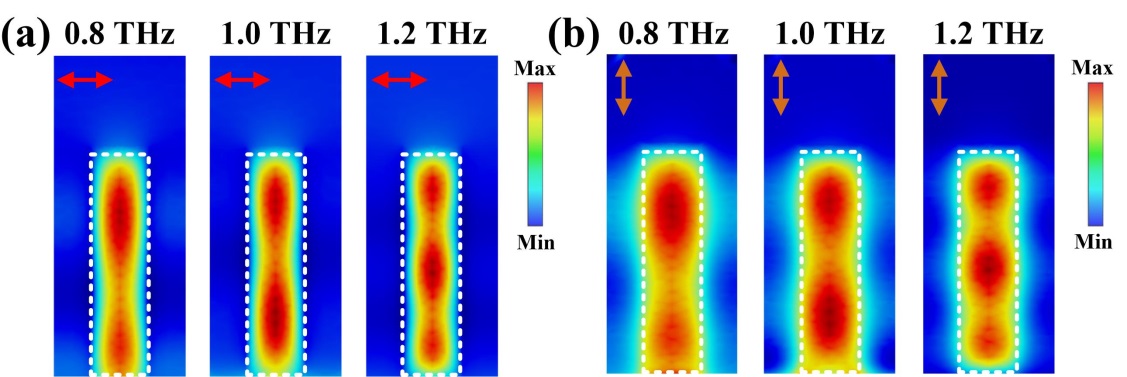


**Figure S1.** The magnetic field distribution at *x*-*z* plane with (a) *x*-polarization, (b) *y*-polarization incident waves. The white dashed lines represent the outline of the meta-atom.

To reveal the light confinement ability of the meta-atoms to incident THz waves, the magnetic field distribution of a circle cross-section meta-atom with radius of 24 μm is simulated after interacting with *x*- and *y*-polarization incident waves, as shown in **Figure S1**. Obviously, both *x*- and *y*-polarization incident waves are entirely confined within the meta-atom at 0.8, 1.0, and 1.2 THz, indicating that it possesses the phase modulation capability of a waveguide-type structure and there is no coupling between adjacent meta-atoms. Therefore, when arranging the spatial position of the meta-atoms, their phase modulation ability will not be affected by adjacent structures.

**Supplementary Section 3:** **The structural parameter variation range of the meta-atoms**

In this part, the change of dimension parameters for six types of meta-atoms is thoroughly discussed. For the circle cross-section meta-atom (Type-I), the transmission and phase responses can be controlled by changing its radius *r*_1_ with the variation range setting as *r*_1_ ∈ [6 μm, 42 μm] and the interval as 0.3 μm. For the ring-shaped cross-section meta-atom (Type-Ⅱ), by changing its outer diameter *r*_2_ and inner diameter *r*_3_ with their respective variation ranges setting as *r*_2_ ∈ [24 μm, 42 μm] and *r*_3_ ∈ [12 μm, 30 μm], and the interval being both 0.3 μm and meeting *r*_2_ – *r*_3_ ≥ 12 μm simultaneously, the transmission and phase responses can be controlled. For the cross-shaped cross-section meta-atom (Type-III), the transmission and phase responses can be controlled by changing its long axis *d*_1_ and short axis *d*_2_ with their respective variation ranges setting as *d*_1_ ∈ [36 μm, 84 μm] and *d*_2_ ∈ [12 μm, 60 μm], and the interval being both 0.6 μm and meeting *d*_1_ – *d*_2_ ≥ 24 μm simultaneously. For the meta-atom with cross hole-shaped cross-section (Type-IV), with the side length of the largest square fixed as 84 μm, the transmission and phase responses can be controlled by changing the side length *d*_3_ and short axis *d*_4_ with their respective variation ranges setting as *d*_3_ ∈ [36 μm, 60 μm] and *d*_4_ ∈ [12 μm, 36 μm], and the interval being both 0.6 μm and meeting *d*_3_ – *d*_4_ ≥ 24 μm simultaneously. For the meta-atom with square-shaped cross-section (Type-V), the transmission and phase responses can be controlled by changing the side length *d*_5_ with the variation ranges setting as *d*_5_ ∈ [12 μm, 84 μm] and the interval as 0.6 μm. For the hole-shaped cross-section meta-atom (Type-VI), with the side length of the largest square fixed as 84 μm, the transmission and phase responses can be controlled by changing the side length *d*_6_ with the variation ranges setting as *d*_6_ ∈ [12 μm, 60 μm] and the interval as 0.6 μm.

**Supplementary Section 4: The optimal design method of the achromatic metalens**

To obtain the optimal target focusing phase for the achromatic metalens at each sampling frequency, an additional phase profile *C*(*f*) is introduced into the hyperbolic focusing phase, as shown in **Figure S2**. Here the *f*_1_, *f*_5_, and *f*_9_ resprent the working frequency at 0.8, 1.0, and 1.2 THz, respectively. Obviously, the additional phase term *C*(*f*) enables the hyperbolic focusing phase translating in the longitudinal directon and thereby meets the matching relationship between the meta-atoms and the target focusing phase of nine discrete frequency points. It is worth noting that for each sampling frequency point, the relative phase at different spatial position remains unchanged, thus it will not affect the target spatial position of the focus. Unlike the continuous wavelength achromatic metalens design method, the discrete multi-wavelength achromatic design does not need to consider the linear relationship between phase and frequency, and effectively reduces the design difficulty. Moreover, the sampling interval of this method is relatively small, amounting to only 5% for the central frequency, which means the achromatic focusing can be realized across the working frequency range because of the focusing phase at each discrete frequency point having a certain working bandwidth.


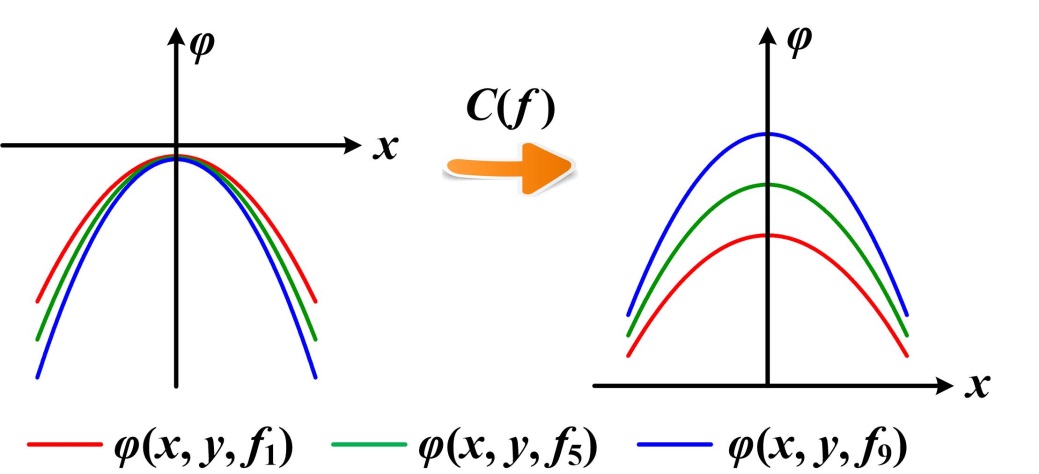


**Figure S2.** The optimal design of the discrete multi-wavelength achromatic metalens.

**Supplementary Section 5: The workflow diagram of PSO algorithm and the convergence curve**

As a leading global optimization algorithm in machine learning, particle swarm optimization (PSO) algorithm, originating from the study of bird predation behavior, has the advantages of easy implementation, high accuracy, and fast convergence [3]. The PSO algorithm initializes a population of random solutions (particles) and iteratively refines them. Each particle’s movement is guided by its own historical best position (individual extremum) and the best position discovered by the entire swarm (global extremum), enabling an efficient search for the global optimum. By continuously updating the speed and position of particles in the search space, the final result is output when the optimization result meeting the indicators or completes the iteration number. The detailed algorithm process is shown in **Figure S3a**. During the iterative optimization process, the additional phase term *C*(*f*) is set as position vector, and Δ*Φ_d_* is regarded as fitness value for the global search. By performing multiple iteration calculations until the curve converges, the optimal *C*(*f*) output can be obtained. **Figure S3b** shows the convergence curve of PSO algorithm after 600 iteration calculations, demonstrating the wavefront phase difference function Δ*Φ_d_* continuously decreases until convergence with the number of iterations increasing.


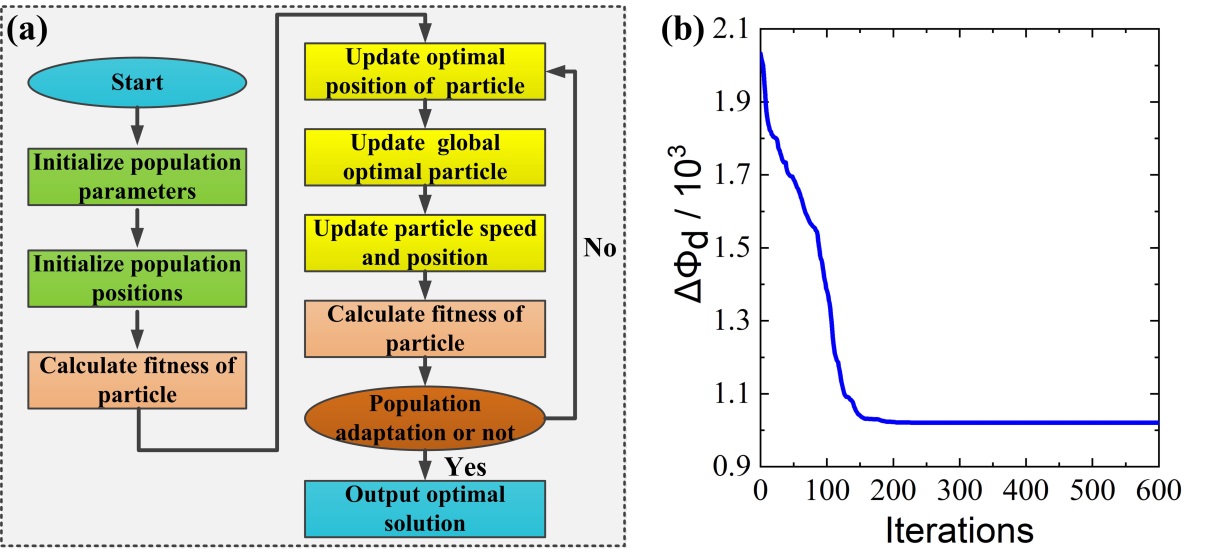


**Figure S3.** (a) The workflow diagram of the PSO algorithm. (b) The convergence curve.

**Supplementary Section 6: Target phase optimization at the sampling frequencies of the metalens**


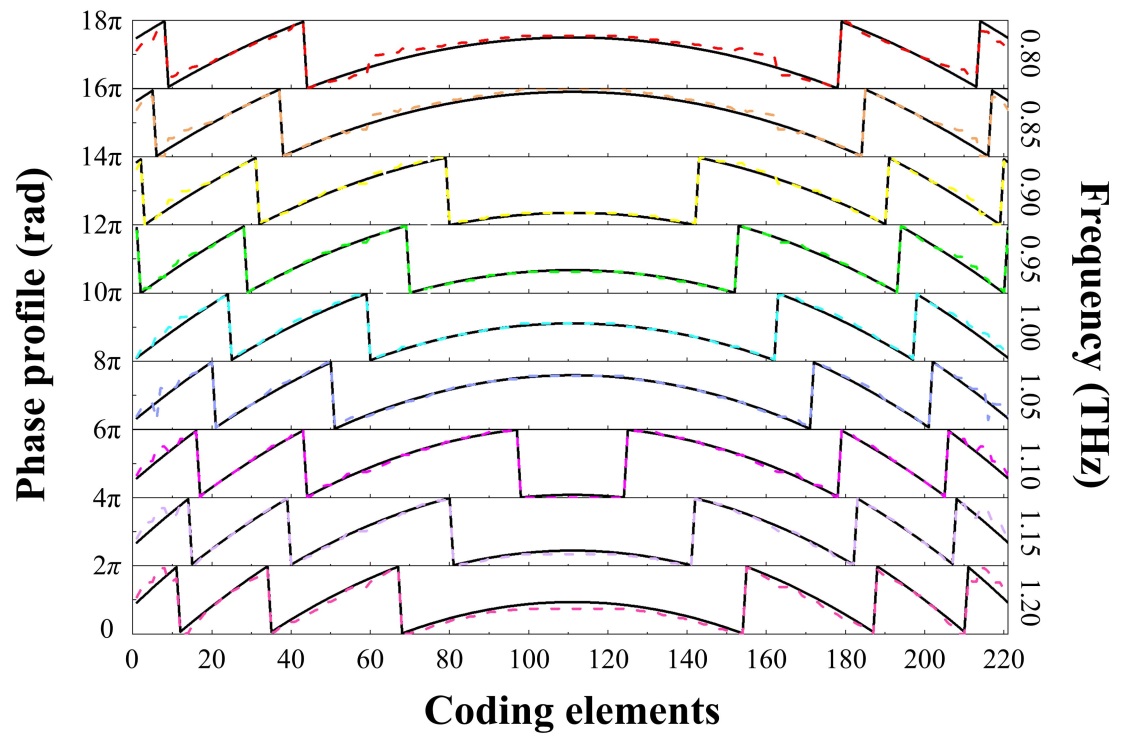


**Figure S4.** The target phase distribution (solid line) and the actual phase distribution (dotted line) of the metalens at each frequency point.

The designed metalens comprises 221 meta-atoms along its diameter direction and its focal length is set as 8 cm. The additional phase term *C*(*f*) can be obtained by multiple iterations of calculation, thereby obtaining the optimal focusing phase distribution at each sampling frequency point. As the number of iterations increasing, the phase difference between the target phase and the actual phase continuously decreases until convergence. As shown in **Figure S4**, the target focusing phases at sampling frequencies are shown as the black solid lines, while the actual phase distributions are indicated by the dashed lines with different colors. Obviously, the actual phase of each sampling frequency point can be perfectly matched with the target focusing phase of the corresponding frequency point. Due to the limited range of dispersion in phase distribution of the meta-atoms, the small phase deviation is introduced at edge position of the metalens, but it has small impact on its performance.

**Supplementary Section 7: The meta-atoms arrangement diagram of the metalens**

To arrange the proposed metalens, the one-to-one correspondence relationship between the optimized target phase and the meta-atoms is used to determine the type and geometric parameter of meta-atom at each spatial position (*x*, *y*), as shown in **Figure S5**. In the enlarged partial image, the red line represents solid structures and the black line represents hollow structures. It can be observed that the designed metalens simultaneously contains six different rotationally symmetric meta-atoms, providing rich design freedom and realizing a large-aperture polarization-independent achromatic function. Subsequently, the metalens can be fabricated by this layout file.


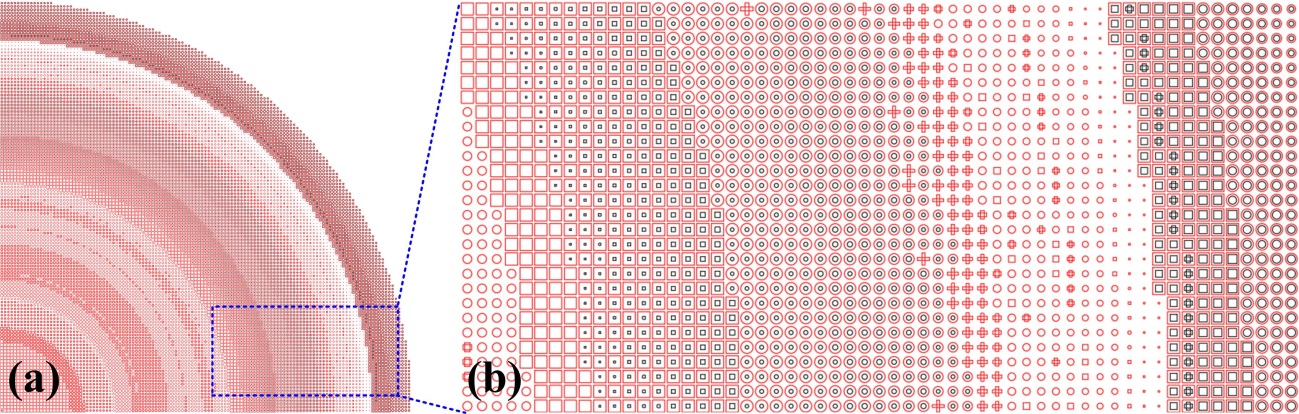


**Figure S5.** The arrangement diagram of the metalens. (a) Quarter area. (b) Partial enlarged drawing.

**Supplementary Section 8: The fabrication process of the metalens**

The fabrication processes of the designed metalens is shown in **Figure S6**, which mainly consists of the following steps: Silicon wafer cleaning and polishing, Metal chromium deposition, Photoresist spin coating, Ultraviolet lithography, Deep reactive ion etching, and Sample cleaning. Particularly, due to the large aspect ratio of the designed meta-atoms, multiple rounds of etching steps are required. During the etching process, etching gas sulfur hexafluoride (SF_6_) and passivation gas octafluorocyclobutane (C_4_F_8_) must be alternately used to protect the vertical side walls of the meta-atoms.


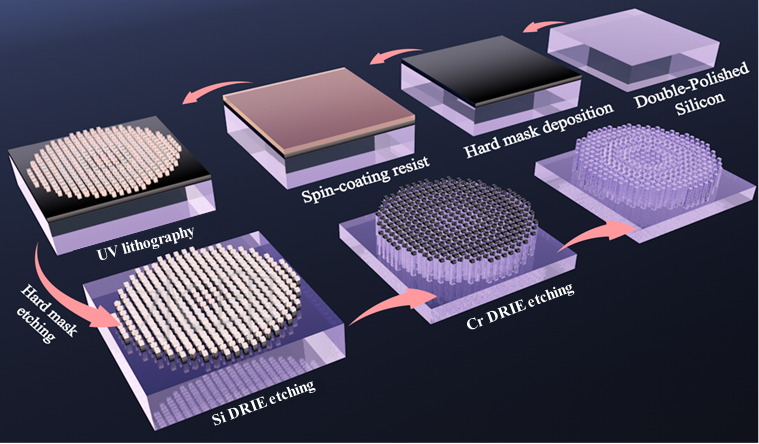


**Figure S6.** Flowchart of the fabrication process for the metalens.

**Supplementary Section 9: The working principle of THz near-field scanning microscopy system**


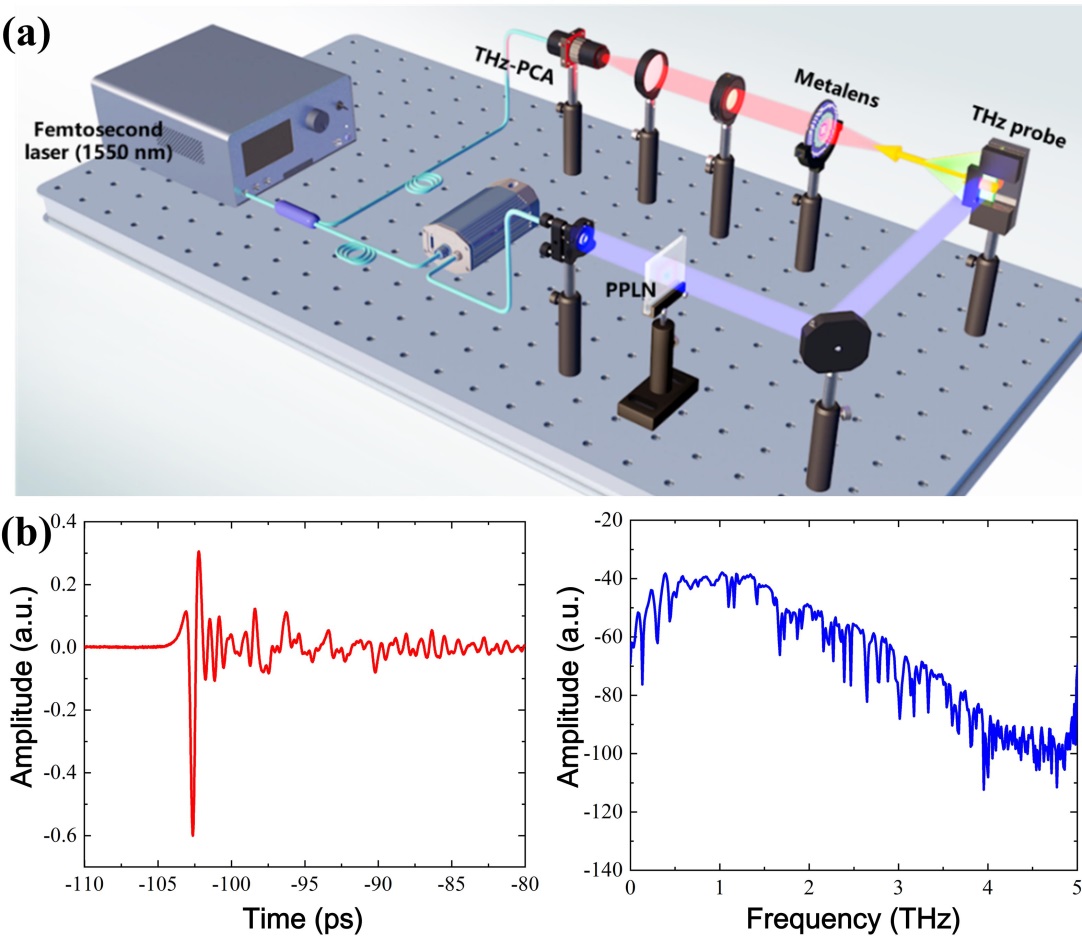


**Figure S7.** (a) Schematic of the experiment system. (b) Time-domain and frequency-domain THz signals.

**Figure S7a** is the optical path diagram of experimental system. Firstly, the optical pulses output by femtosecond laser with central wavelength of 1550 nm are split by a beam splitter into two beams. One of the femtosecond laser pulses is irradiated onto the THz photoconductive antenna, thereby generating THz waves and vertically irradiate on the sample with linear polarization. The other femtosecond laser pulse is first frequency doubled through lithium niobate crystal PPLN and then irradiated onto the THz near-field probe for achieving high-resolution detection of THz signals. The effective spectrum of this system is from 0.1 to 2.5 THz with a minimum spatial resolution of 20 μm. Both the probe and the sample are mounted on a 3D motorized stage to scan the 3D electric field distribution. **Figure S7b** shows the THz time-domain and frequency-domain signals generated by this system.

**Supplementary Section 10: The** **simulated performance** **of the metalens under *x*-polarized incident THz waves**

The simulated FWHMs at sampling frequency points are calculated in **Figure S8a**, which are 1.13 mm, 1.07 mm, 1.04 mm, 1.00 mm, 0.96 mm, 0.93 mm, 0.90 mm, 0.86 mm, and 0.81 mm, respectively. The focusing efficiency are also calculated in **Figure S8b**, ranging from 46.95% (minimum value at 0.80 THz) to 58.73% (maximum value at 1.00 THz) with the average focusing efficiency as 53.97%.


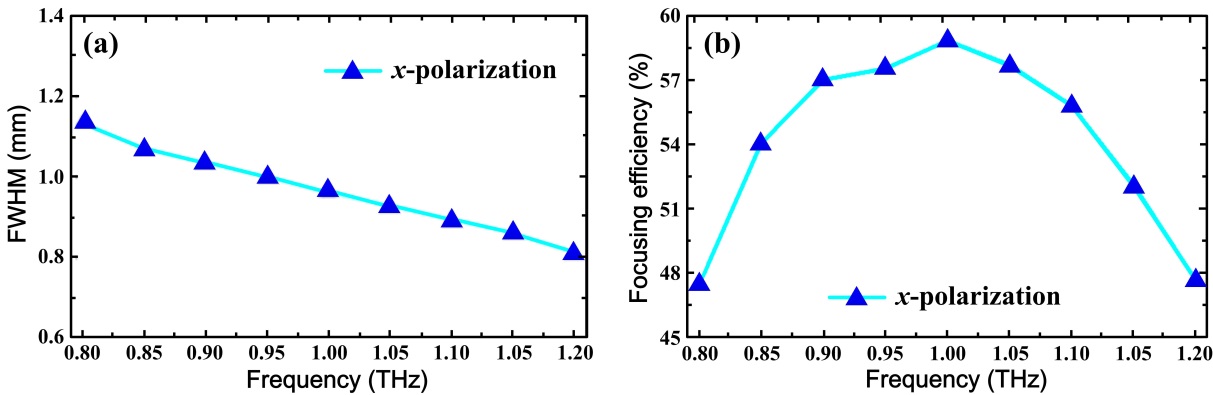


**Figure S8.** The simulated results under *x*-polarized incident THz waves. (a) FWHM. (b) Focusing efficiency.

**Supplementary Section 11: The simulated performance of the** **metalens under *y*-polarized incident THz waves**

Similarly, the simulated FWHMs at sampling frequency points under *y*-polarized incidence are calculated in **Figure S9a**, which are basically consistent with the results of *x*-polarized incidence waves. The focusing efficiency are calculated ranging from 46.02% (minimum value at 0.80 THz) to 57.57% (maximum value at 1.00 THz) with the average focusing efficiency as 52.88%, as shown in **Figure S9b**.


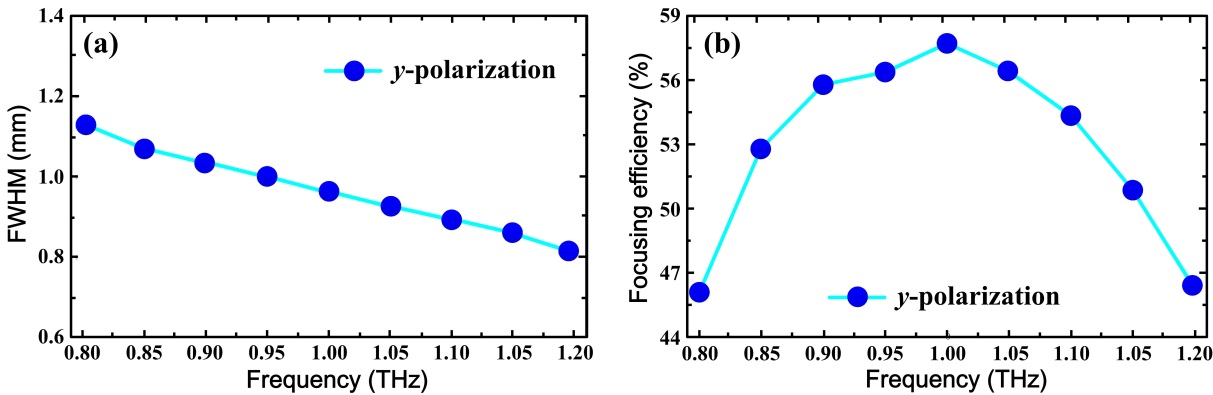


**Figure S9.** The simulated results under *y*-polarized incident THz waves. (a) FWHM. (b) Focusing efficiency.

**Supplementary Section 12: The achromatic imaging for sample with 1.8 mm gap**

**Figure S10** shows the achromatic imaging results for the U-shaped pattern with 1.8 mm gap. Clearly, the sample can be recognized under broadband and low frequencies including 0.8, 0.9, and 1.0 THz. However, it cannot be identified at high frequencies including 1.1 and 1.2 THz, which is due to the significantly decreased focusing efficiency, the reduction of THz power at high frequencies, the experimental error, and the diffraction effect. The imaging quality can be further enhanced via enlarging NA and focusing efficiency of the metalens, increasing power of THz waves, which relying on innovative design methods, advanced processing technology, and high power THz-TDS system.


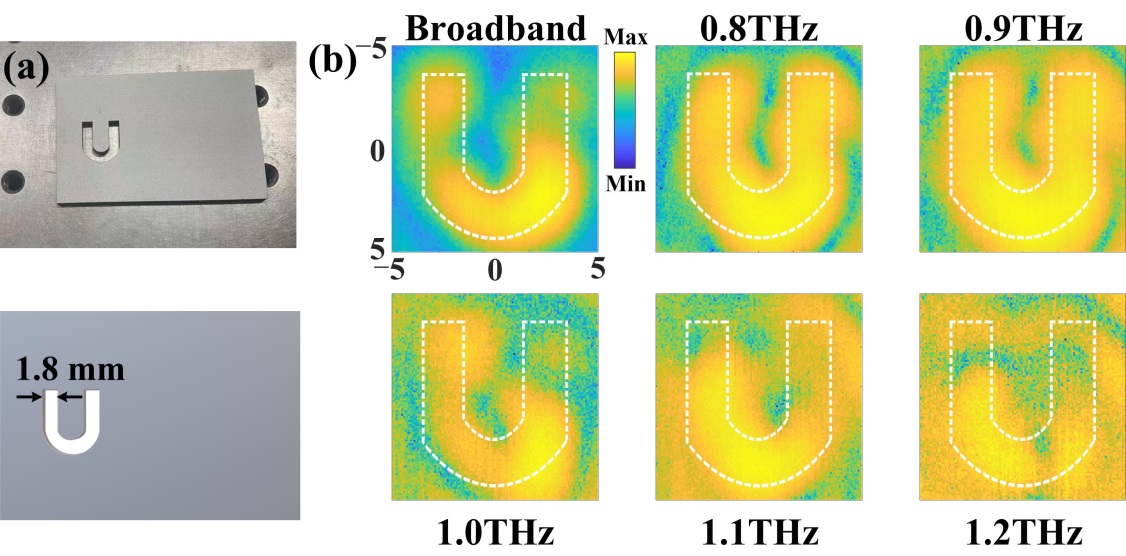


**Figure S10.** The achromatic imaging results of 1.8 mm gap sample. (a) U-shaped object. (b) The measured achromatic images, the white dashed line representing the outline of the sample.

**References**

1. N. Yu, P. Genevet, M. A. Kats, F. Aieta, J.-P. Tetienne, F. Capasso, and Z. Gaburro, “Light propagation with phase discontinuities: generalized laws of reflection and refraction,” *Science* 334, 333 (2011).
2. S. Shrestha, A. C. Overvig, M. Lu, A. Stein, and N. Yu, “Broadband achromatic dielectric metalenses,” *Light: Science & Applications* 7, 85 (2018).
3. R. Thangaraj, M. Pant, A. Abraham, and P. Bouvry, “Particle swarm optimization: Hybridization perspectives and experimental illustrations,” *Applied Mathematics and Computation* 217, 5208 (2011).
